# Premature mortality due to four main non-communicable diseases and suicide in Brazil and its states from 1990 to 2019: A Global Burden of Disease Study

**DOI:** 10.1590/0037-8682-0328-2021

**Published:** 2022-01-28

**Authors:** Ewerton Cousin, Maria Inês Schmidt, Caroline Stein, Érika Carvalho de Aquino, Ellen de Cassia Dutra Pozzetti Gouvea, Deborah Carvalho Malta, Mohsen Naghavi, Bruce B. Duncan

**Affiliations:** 1 University of Washington, Institute for Health Metrics and Evaluation, Seattle, WA, United States.; 2 Universidade Federal do Rio Grande do Sul, Programa de Pós-Graduação em Epidemiologia, Porto Alegre, RS, Brasil.; 3 Universidade Federal do Rio Grande do Sul, Faculdade de Medicina, Departamento de Medicina Social, Porto Alegre, RS, Brasil.; 4 Ministério da Saúde, Coordenação-Geral de Vigilância de Doenças e Agravos não Transmissíveis, Departamento de Análise de Saúde e Vigilância de Doenças Não Transmissíveis, Brasília, DF, Brasil.; 5 Universidade Federal de Minas Gerais, Escola de Enfermagem, Departamento de Enfermagem Materno-Infantil e Saúde Pública, Belo Horizonte, MG, Brasil.

**Keywords:** Mortality, Noncommunicable diseases, Global Burden of Disease, Brazil

## Abstract

**INTRODUCTION:**

The goal of reducing the burden of non-communicable diseases (NCDs) requires close monitoring. Our objective is to characterize the decline of premature NCD mortality in Brazil based on Global Burden of Diseases (GBD) Study 2019 estimates.

**METHODS:**

We used GBD 2019 data to estimate death rates of the four main NCDs - cardiovascular diseases, neoplasms, diabetes, and chronic respiratory diseases. We estimated the unconditional probability of death between ages 30 to 69, as recommended by the World Health Organization, as well as premature crude- and age-standardized death rates and disability-adjusted life years (DALYs) lost for these conditions. We also estimated trends in suicide (self-harm) death rates.

**RESULTS:**

From 2010 to 2019, the age-standardized unconditional probability of premature death declined -1.4%/year (UI: -1.7%;-1.0%) . Age-standardized death rates declined -1.5%/year (UI: -1.9%; -1.2%), and crude death rates -0.6%/year (UI: (-1.0%; -0.2%). Level of development correlated strongly with the rate of decline, with greatest declines occurring in the Southeast, Center West and South regions. Age-standardized mortality from self-harm declined, most notably in the elderly.

**CONCLUSIONS:**

Premature mortality due to the main NCDs has declined from 1990 in Brazil, although at a diminishing rate over time. The unconditional probability of death and the age-standardized mortality rate produced similar estimates of decline for the four main NCDs, and mirror well decline in mortality from all NCDs. Declines, especially more recent ones, fall short of the international goals. Strategic public health actions are needed. The challenge to implement them will be great, considering the political and economic instability currently faced by Brazil.

## INTRODUCTION

In 2011 the Brazilian Ministry of Health, following the lead of the World Health Organization (WHO), launched a plan to confront the challenge of non-communicable diseases (NCDs), with a goal of reducing NCD mortality annually by 2% up to 2025^1^. In 2013, the WHO proposed the use of a specific metric for monitoring, the unconditional probability of premature death from four main disease groups - cardiovascular diseases, neoplasms, diabetes, and chronic respiratory diseases. Premature was defined, as occurring over a 40 years period, starting at age 30 and terminating at age 69^2^. Since Brazil launched its NCD action plan prior to the establishment of this definition^3^, used a similar metric - mortality under 70 for the same four groups of diseases, subsequently defined as age-standardized mortality occurring over the same age range from 30-69 authorities chose 2010 as the baseline year.

In 2015, the United Nations (UN) launched the Sustainable Development Goals (SDGs), in which the principal indicator of progress against the NCDs is SDG target 3.4.1 - to reduce premature mortality from NCDs by a third from 2015 to 2030^4^ ^,^ ^5^. Additionally, more recently the WHO expanded the scope of its focus from four to five NCD groups by including mental health, and indicated as its SDG target 3.4.2 the suicide mortality rate^6^.

Considering that the Brazilian Ministry of Health is now in the process of launching a continuation of its plan to confront the NCDs^7^, evaluation of progress in controlling the NCDs as measured by each of these indicators is indicated. Data from the Global Burden of Disease (GBD) 2019 Study^8^ ^,^ ^9^, which has systematically received Brazilian mortality data and adjusted it for underreporting and ill-defined causes, offers an excellent platform to evaluate progress in confronting the NCDs as measured by these different metrics.

A relevant additional question, in this process, is the adequacy of these metrics considering broader goals of decreasing premature mortality and overall disease burden caused by all of the NCDs, and additionally, considering the NCD burden at all ages.

Our objective is thus to characterize the decline in premature mortality due to the four main NCDs, contrasting progress as measured by the indicators adopted by the WHO and the Brazilian Ministry of Health. Additionally, we will compare results of these two indicators against others measuring NCDs trends more broadly and present the GBD estimate of deaths in Brazil from suicide.

## METHODS

We used the estimates from the Global Burden of Diseases (GBD) Study 2019. The GBD applies complex methods to estimate mortality which have been described in detail elsewhere^8^ ^,^ ^9^. To estimate NCD deaths in Brazil the GBD mapped International Classification of Disease (ICD) 9 and 10 codes from the Brazilian mortality information system (Sistema de Informações sobre Mortalidade in Portuguese, or SIM) to disease causes as defined by the GBD.  Supplementary Table 1 presents the list of ICD-9 and 10 codes for each cause of death^9^. Adjustment for incompleteness in death reporting was based on five death distribution methods along with the evaluation of under-5 vital registration completeness^8^ ^,^ ^10^. Deaths assigned in SIM to causes that cannot be the underlying cause of death or are inadequately specified (e.g. sepsis and heart failure), denominated garbage codes, were reassigned to the most probable underlying cause of death using redistribution algorithms^9^. The GBD then applies Cause of Death Ensemble modeling (CODEm), a tool which combines results from an ensemble of different modelling approaches, to estimate death rates^9^.

To calculate the unconditional probability of dying between ages 30 to 70 (up to and including age 69) from the four main disease groups, we used the formula for this metric recommended by the WHO^11^ ^,^ ^12^.

The probability of death (_5_q_x_) in each 5-year age range is obtained from the 5-year death rates (_5_M_x_):

$${\;}_{5}q_{x}=\frac{5M_{x}*5}{1+5M_{x}*2.5}$$

where _5_M_x_ is the age-specific mortality rate as estimated by the GBD for each five-year age group between 30 and 70, being:

$$5M_{x}=\frac{\text{Total deaths from four NCD causes between exact age}x\text{and exact age}x+5}{\text{Total population between exact age}x\text{and exact age}x+5}$$

Then the uncondional probability of death from age 30 to 70 (_40_q_30_) is determined as follows:

$${}_{40}q_{30}=1-\prod_{x=30}^{65}\;\left(1-{}_{5}q_{x}\right)$$

Additionally, we calculated the crude and age-standardized death rates and disability-adjusted life years (DALYs) lost for the four main NCDs from age 30 to 69. The DALYs metric is a single measure that accounts for both premature mortality and morbidity, being estimated as the sum of years of life lost (YLLs) due to premature mortality and years lived with disability (YLDs)^9^. All age-standardized estimates were calculated using the GBD world population age standard^8^.

The annualized rate of change, is calculated using the formula below^8^:

$$\beta =\frac{ln\left(\frac{40q_{30}y+t}{{\;}_{40}q_{30}y}\right)}{t}$$

with the a with annual average change in 40q30 = *e* ^β^

We extrapolated rates of age-standardized mortality and the unconditional probability of dying applying the annualized rate of change from 2010-2019 and from 2015-2019 to the estimates of these metrics in 2019, out to the years 2025 and 2030 to estimate the level of these metrics at the end of the periods used in the WHO goals.

For the extrapolations was used the formula below:

$$projection{\;}_{2019+t}=exp((ln(arc)*t)+In(x))$$

Where arc = annualized rate of change, t = number of years projected, x = value at time 2019. 

We also calculated the crude death rates due to self-harm, the term used by the GBD to characterize suicide, to produce the UN´s SDG suicide mortality rate metric, defined as the number of self-harm deaths in a year, divided by the population, and multiplied by 100,000^6^. We also calculated age-standardized self-harm rates to describe trends.

We report the 95% uncertainty interval (UI) for each estimate based on the 25th and 975th ordered values of 1000 draws of the posterior distribution. GBD analyses were conducted with Python version 3.6.2, Stata version 13, and R version 3.5.0. The analyses specific to this study were done with R version 3.6.

In comparing metrics for the different Brazilian states, we used the socio-demographic index (SDI) as an indicator of level of development^8^. SDI is a composite index, ranges from 0 to 1, measuring per capita income, fertility, and education, and reveals the differences in the socio-demographic development of each state^8^. We fitted linear regression lines and used the coefficient of determination to express the correlation of level of development with the 2019 level of the metrics and also with their change over time. We evaluated the statistical significance of these differences testing whether the inclination of these lines differed from zero.

## RESULTS

**FIGURE 1:**
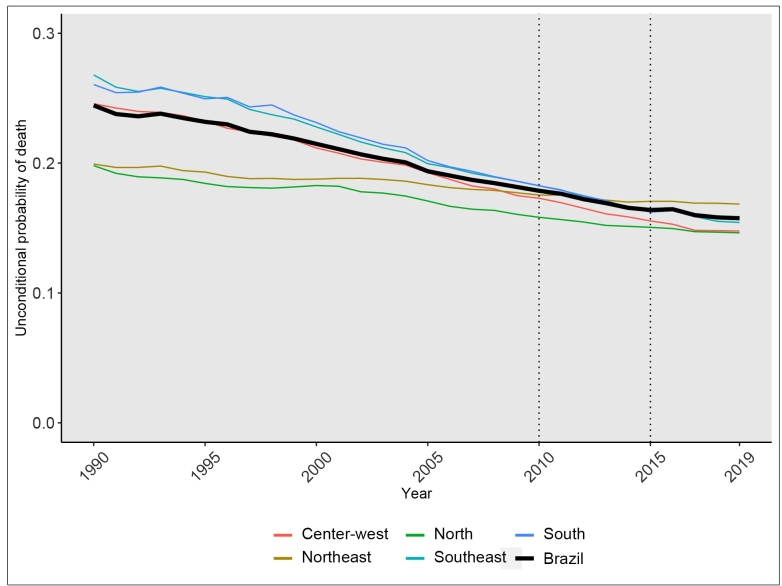
Unconditional probability of death between ages 30 and 69 years due to cardiovascular diseases, neoplasms, chronic respiratory diseases, and diabetes for Brazil and its regions, 1990-2019.

The unconditional probability of death due to the four main NCDs declined -1.6% [Uncertanty interval (UI): -1.7; -1.5] annually in Brazil from 1990 to 2010, decreasing over the whole period from 24% (UI: 24;25) in 1990 to 18% (UI: 17.5;18.2) in 2010. The rate of decline was less - -1.4%/year (UI: -1.7; -1.0) - between 2010-2019 and even less, -1.0%/ year (-1.7%; -0.2) from 2015 onward. Analyzing these trends by regions, we found that states in the Southeast region had a higher rate of decrease in the period (-1.9%/ year (UI: -2.0; -1.8) annually up to 2010 and -1.9%/ year (UI: -2.4; -1.3) from 2010 to 2019). On the other hand, states in the Northeast region showed the lowest annualized percentage change in the unconditional probability of death in the period, -0.6% (UI: -0.9; -0.4) between 1990-2010 and -0.4% (UI: -1.2; 0.3) from 2010-2019 (Figure 1 **,** Table 1). 

**TABLE 1:** Different measures in mortality and disease burden from non-communicable diseases (NCDs) in Brazil, and their trends from 1990 and from the 2010 baseline for action plans to confront the NCDs, as calculated from GBD 2019 data, unless stated otherwise.

		1990	2010	2019	Annualized percent change 1990-2010	Annualized percent change 2010-2019
	**Disease Burden, Ages 30-69**					
	Uncond prob. death	0.244 (0.240;0.249)	0.179 (0.175;0.182)	0.158 (0.152;0.162)	-1.6% (-1.7;-1.5)	-1.4% (-1.7;-1.0)
	Crude death rate	449.1 (440.0;458.6)	339.0 (331.6;345.2)	321.8 (310.4;332.7)	-1.4% (-1.5;-1.3)	-0.6% (-1.0;-0.2)
	Age-standardized death rate	532.1 (521.3;543.1)	371.6 (363.2;378.5)	323.0 (311.7;334.0)	-1.8% (-1.9;-1.7)	-1.5% (-1.9;-1.2)
	Uncond. prob. death, WHO^11^		0.178	0.155		-1.50%
	Crude DALYs	16348 (15844;16840)	12550 (12098;13039)	11844 (11282;12457)	-1.3%(-1.4;-1.2)	-0.6% (-1.0;-0.3)
	Age-standardized DALYs	18710 (18,132;19,260)	13392 (12,914;13,911)	11845 (11,284;12,459)	-1.7% (-1.8;-1.5)	-1.4% (-1.7;-1.0)
**Unconditional probability of death, ages 30-69**	**Region - Four Main NCDs**					
	North	0.198 (0.186;0.210)	0.158 (0.149;0.167)	0.146 (0.138;0.154)	-1.1% (-1.5;-0.7)	-0.9% (-1.7;.0.0)
	Northeast	0.199 (0.192;0.207)	0.175 (0.168;0.183)	0.168 (0.59;0.178)	-0.6% (-0.9;-0.4)	-0.4% (-1.2;.03)
	Center-West	0.246 (0.230;0.267)	0.173 (0.165;0.181)	0.148 (0.137;0.159)	-1.7% (-2.2;-1.4)	-1.7% (-2.7;-0.8)
	Southeast	0.268 (0.262;0.274)	0.183 (0.179;0.186)	0.154 (0.147;0.162)	-1.9% (-2.0;-1.8)	-1.9% (-2.4; -1.3)
	South	0.260 (0.257;0.263)	0.183 (0.179;0.186)	0.159 (0.149;0.168)	-1.8% (-1.9;-1.7)	-1.6% (-2.2;-0.9)
	**Cause**					
	Cardiovascular diseases	0.147 (0.144;0.150)	0.089 (0.87;0.091)	0.074 (0.072;0.077)	-2.5% (-2.6;-2.4)	-2.0% (-2.4;-1.6)
	Neoplasm	0.08 (0.078;0.083)	0.073 (0.071;0.074)	0.066 (0.064;0.069)	-0.5% (-0.6;-0.3)	-1.0% (-1.3;-0.6)
	Chronic respiratory diseases	0.02 (0.018;0.021)	0.013 (0.013;0.014)	0.012 (0.011;0.013)	-1.9% (-2.1;-1.6)	-1.6% (-2.2;-1.0)
	Diabetes	0.018 (0.017;0.018)	0.015 (0.014;0.015)	0.014 (0.013;0.014)	-0.9% (-1.2;-0.7)	-0.9% (-1.4;-0.3)
**Crude death rate**	**Cause**					
	Cardiovascular diseases	255.9 (250.6;261.6)	160.0 (256.3;163.1)	144.7 (139.0;150.2)	-2.3% (-2.5;-2.2)	-1.1% (-1.5;-0.7)
	Neoplasm	136.7 (133.3;142.3)	133.5 (130.4;136.6)	131.7 (126.8;136.6)	-0.1% (-0.3;0.0)	-0.1% (-0.5;.03)
	Chronic respiratory diseases	29.0 (27.2;30.4)	21.5 (20.6;22.7)	20.7 (19.5;22.4)	-1.5% (-1.7;-1.2)	-0.4% (-1.1;0.2)
	Diabetes	27.5 (26.6;28.4)	24.0 (23.2;24.8)	24.7 (23.5;25.7)	-0.7% (-0.9;-0.5)	0.3% (-0.2;0.8)
**Age-standardized death rate**	**Cause**					
	Cardiovascular diseases	303.0 (296.5;309.8)	175.6 (171.3;179.0)	145.3 (139.5;150.9)	-2.7% (-2.8;-2.6)	-2.1% (-2.5;-1.6)
	Neoplasm	160.8 (156.7;167.3)	145.1 (141.6;148.6)	132.0 (127.1;137.0)	-0.5% (-0.7;-0.4)	-1.0% (-1.4;-0.6)
	Chronic respiratory diseases	35.4 (33.1;37.1)	24.2 (23.1;25.6)	20.9 (19.6;22.7)	-1.9% (-2.1;-1.6)	-1.6% (-2.2;-1.0)
	Diabetes	33.0 (32.0;34.2)	26.8 (25.9;27.6)	24.8 (23.7;25.9)	-1.0% (-1.2;-0.8)	-0.9% (-1.4;-0.3)

**Uncond. prob:** Unconditional probability.

**FIGURE 2:**
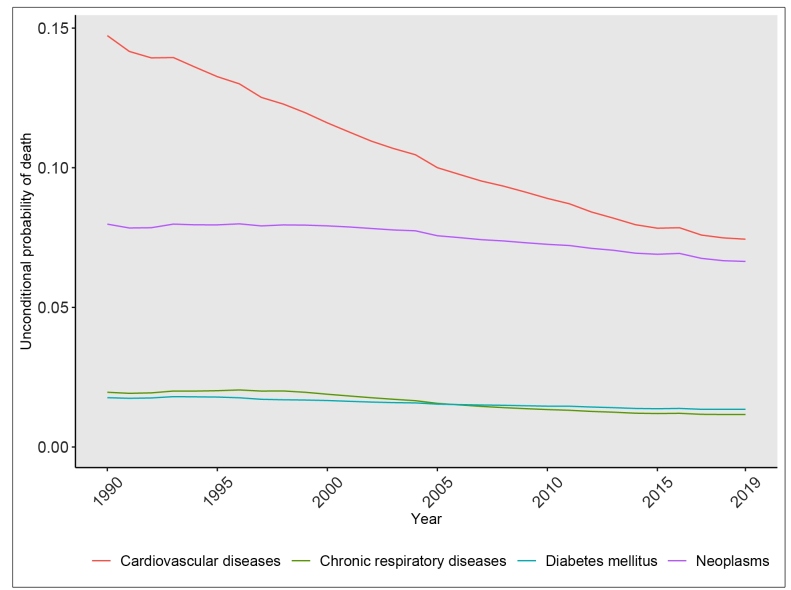
Trends in the unconditional probability of death from 30 to 69 years of age due to the each of the four main non-communicable diseases, 1990-2019.

Cardiovascular diseases were the cause with the highest decrease over the period, moving from a 14.7% (UI: 14.4; 15.0) probability of death due to these diseases in 1990 to a 7% (UI: 7.2; 7.7) one in 2019, with an annualized change of -2%/ year (UI: -2.4; -1.6) from 2010 to 2019. Chronic respiratory diseases showed a major, though slightly smaller annualized decrease, -1.6%/ year (UI: -2.2; -1.0), during this period. The annualized decrease for neoplasms and diabetes was less pronounced, -1%/ year (UI: -1.3;-0.6) and -0.9%/ year (UI: -1.4; -0.3) from 2010 to 2019, respectively (Figure 2 and Table 1).

Supplementary Figure 1 and Table 1 compare trends in the unconditional probability of death from the four main NCDs versus age-standardized and crude death rates due to the same causes. In this figure, the standard population is that of 2019, so that crude and age-standardized rates are equal in 2019. We used two y-axis scales to permit a comparison of percent decline between the three metrics, placing the axis of the unconditional probability of death so that this metric was at the same level in 2019 of the other two metrics. This format,, which allows a uniform vision of the decline of the three metrics, shows that the crude death rate shows the least pronounced decrease (-0.6%/ year (UI: -1.0; -0.2) from 2010) and age-standardized mortality the most pronounced (-1.5%/ year (UI: -1.9; -1.2) from 2010). From 2015 onwards, age-standardized mortality decreased only 1.1%/year (-1.9; -0.2). In 2018, the crude mortality reached its current nadir, 320.0/100,000, being slightly up from that in 2019.

Table 1 also shows the trends in the crude and age-standardized rates of DALYs for the four main NCDs in Brazil from 1990 to 2019. It demonstrates declines in DALYs equal to or slightly less than in deaths: the annualized percentage change in crude and age-standardized DALYs rates, for ages 30-69 were -0.6%/ year (UI: -1.0; -0.3) and -1.4%/ year (UI: -1.7; -1.0) from 2010 onwards, vs. -0.6% (UI-1.0; -0.2) and -1.5% (UI: -1.9; -1.2) changes in the crude and age-standardized mortality rates. 

Supplementary Figure 2 shows the comparison in the unconditional probability of death from one of the four main NCDs and from all NCDs combined. The probability of death from any NCD is somewhat higher but decreases in parallel with that of the unconditional probability of death from one of the four main NCDs. 

As shown in  Supplementary Table 2, extrapolating to 2025 the rate of decline in age-standardized mortality ages 30-69 over the period 2010-2019 shows a final rate 294.2 (277.8; 310.9)/100,000 deaths, and when extrapolating the rate achieved over the period 2015-2019, a 2025 rate of 302.5 (277.9; 328.1)/100,000. Extrapolating these changes out to 2030 produces final rates of 272.1 (252.3; 293.3)/100,000 and 286.4 (252.2; 324.2)/100,000, respectively. The total percent declines estimated from 2015 to 2030 are 19.3% (25.0; 11.6) and 15.0% (25.1; 3.4)), respectively, both short of the established goal of 33%. Similar extrapolation of the unconditional probability of death ages 30-69 produces similar results. 

**FIGURE 3:**
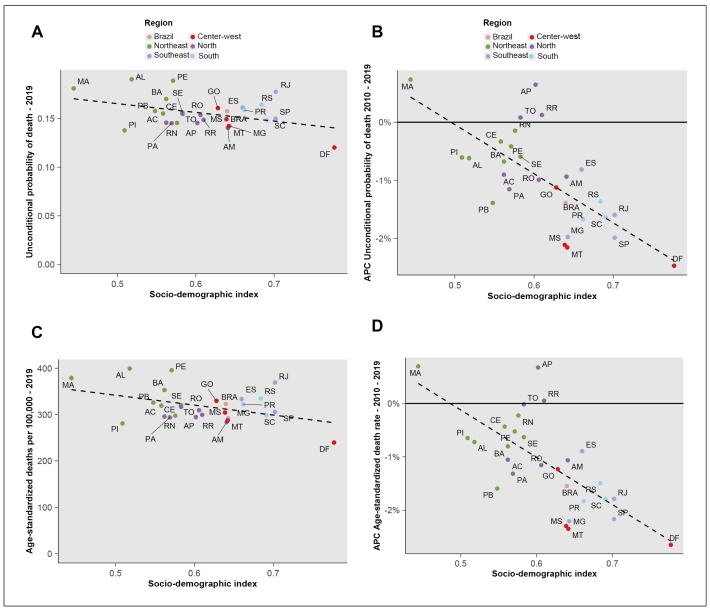
Mortality from the four main non-communicable diseases groups of Brazilian states, 30 to 69 years of age, according to the sociodemographic index of Brazilian states in 2019. Left panels: 2019. Right panels: Annual percentage change (APC) 1990-2019. Top panels: Unconditional probability of death, bottom panels: Age-standardized mortality/100000 population.

The unconditional probability of death and age-standardized mortality rates in 2019 for Brazilian states showed little correlation with their 2019 SDI (r^2^= 0.16, p=0.03 and r^2^=0.17, p=0.03). However, the annualized rate of decline from 2010 to 2019 showed significantly more decrease with higher SDI values for both metrics (r^2^= 0.50, p<0.001 and r^2^=0.48, p<0.001), respectively, demonstrating greater progress in controlling NCD mortality in the more developed states of the Southeast, South, and Center West (Figure 3).

**FIGURE 4:**
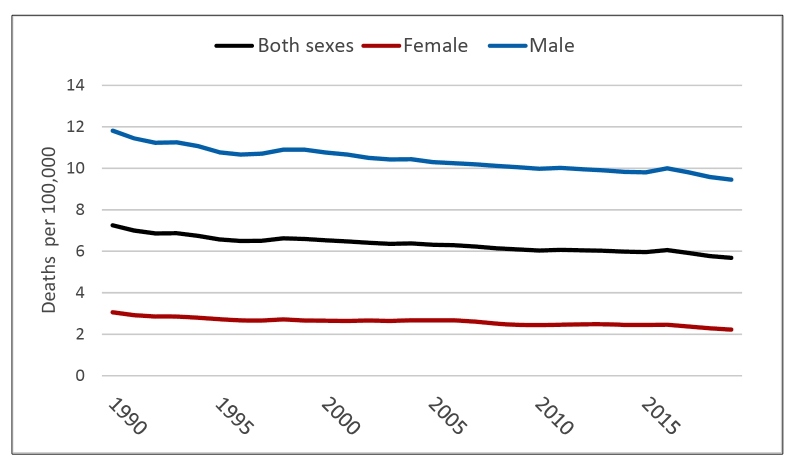
Overall and sex-specific trends in the age-standardized death rate due to self-harm, from 1990 to 2019. All ages, Brazil.

The age-standardized death rate due to self-harm declined 22% from 1990 to 2019 (Figure 4), from 7.25 (7.06; 7.50) deaths/100,000 in 1990 to 5.68 (5.40; 6.19) deaths/100,000 in 2019. Rates were considerably higher in males than females. Rates became appreciable in the 15-19 age range, increased markedly up to 25-29 age range and then slowly climbed thereafter ( Supplementary Figure 3).Rates have been stable over time in the young but have decreased 37% in those age 70 or older ( Supplementary Figure 4).

## DISCUSSION

Brazil has decreased the unconditional probability of premature death due to the four main NCDs approximately 1.4%/year from 2010 to 2019, and the crude and age-standardized rates of premature death from these conditions 0.6%/year and 1.5%/year, respectively, falling short of the Strategic Action Plan´s goal of 2%/year. The trends suggest that total declines considerably less than the WHO´s 2025 goal of a 25% reduction and the SDG 3.4.1´s 2030 goal of a 33% reduction in premature mortality due to the NCDs will be achieved, especially if more recent rates are used. The Southeast, Center West and South regions have seen greater reductions than the North and especially the Northeast, with a strong correlation existing between the level of development and the rate of decline. Age-standardized deaths from suicide have declined over this period, particularly in the elderly.

The unconditional probability of premature death from the four main diseases tracks well with the unconditional probability of premature death from all NCDs, justifying its use internationally and in Brazil, to permit international comparisons. Little difference exists between this WHO metric and the age-standardized mortality metric of the Brazilian Ministry. A potential advantage of the unconditional probability of death metric is its interpretability at the individual level (e.g., a reduction from 15% to 7% in the average Brazilian´s risk of a premature cardiovascular death occurred in Brazil from 1990 to 2019). However, the age-standardized mortality metric to date has declined a bit more, making it a slightly easier target to achieve. As age-standardized mortality weighs the larger younger age strata more heavily while the unconditional probability of death weighs each age strata equally, the greater decline in age-standardized premature mortality shows that declines having been greater in younger adults within the 30-69 year age range.

Over the period, as previously shown with SIM data, the decline has flattened noticeably in cardiovascular and chronic respiratory disease mortality, while increasing somewhat for cancer deaths^13^. Declines in deaths due to diabetes have been stable. The mixed results of controlling lifestyle-determined risk factors such as dietary risks, physical inactivity, harmful alcohol consumption and smoking, coupled with major worsening in the prevalence of overweight and obesity, and with them worsening of hyperglycemia, hypertension and dyslipidemia, have undoubtedly contributed to this flattening^14^.

The more substantial rates of decline in the more developed regions of Brazil, as demonstrated by the correlation between the SDI of states and their declines, suggests that greater attention to NCD prevention is particularly necessary in the poorer North and Northeast regions. Why decline has been worse in these regions can only be speculated. However, greater disease burden from infectious diseases and maternal/child conditions produces a greater double burden of disease requiring health planners to divide their attention to the NCDs with these other conditions. These regions also have undergone a much more rapid nutritional and epidemiologic transition, have lower levels of educational attainment and have less resources in general for public health and healthcare. Greater awareness of those orienting public health measures in the North and Northeast to the rise of the NCD burden and options for its control, given that the GBD estimates that it now causes 60%-70% of all DALYs in the states of these regions, is of major importance^15^.

The data presented demonstrate that suicide is an important public health problem. The determination of suicide is multiple and complex, involving both exogenous determinants - financial problems, disruption of relationships, trauma and exposure to violence and disasters - and endogenous one - family history of suicide, chronic illnesses, mental disorders, and feelings of loneliness and hopelessness^16^. 

Mental disorders play an important role in suicidal behavior. More than 90% of suicide victims are estimated to have had some mental disorder, depression being the most frequent among them^17^. In addition, individuals with mental disorders are eight times more likely to commit suicide^18^. Data from the National Health Surveys of 2013 and 2019 point to an increase in the prevalence of depression diagnosed by mental health professionals in individuals over 18 years of age - from 7.6% (95% CI: 7.2 ; 8.1) in 2013 to 10.2% (95% CI: 9.9; 10.6) in 2019. In 2019, less than half (52.8%) of these individuals reported having received specialized medical care in the previous 12 months, with only 18.9% reporting having received psychotherapy and less than half of both men (43.8%) and women (49, 3%) having received medications^19^ ^,^ ^20^. These data make evident the need to expand and democratize access to mental health in Brazil, as well as the importance of early identification of mental disorders in primary care. 

An estimated annualized rate of decline of 2.67% from 2015 to 2030 would be necessary to achieve the SDG goal^21^, far beyond that currently being achieved. In Brazil, studies evaluating the impacts of the economic crises and the implementation of Constitutional Amendment 95 in 2016, which limits governmental spending on health and education, point to an increase in unemployment^22^, poverty, worsening indicators in health^23^, accompanying reduced investments in health systems and in the social protection system^23^ ^,^ ^24^. Austerity measures increase regional inequalities and social disparities, complicating the achievement of the SDG goals^25^. The disarray within Brazilian society caused by COVID-19 will likely only complicate the situation further.

A continuing decrease in age-standardized measures of NCD mortality is vital to counteract the effect of aging of the population in crude burden - the burden which society in fact suffers. As disease incidence increases dramatically with age, decreases in the age-standardized rate are necessary to avoid growth in the crude rate of NCDs. As can be seen in **Supplemental Figure 1**, crude mortality is no longer decreasing, although tracking of changes over the next few years will be necessary to confirm this stabilization. Thus, the flattening of the declines in the unconditional probability of death and in age-standardized mortality in recent years are of particular concern. Increases in crude NCD disease rates have an enormous impact on quality of life of Brazilians and resources necessary for healthcare in Brazil. 

Brazil is not alone in lagging in the rate necessary to achieve the goal and, in fact, has done better than most nations. The more recent flattening of the decline has been seen throughout the Americas for cardiovascular diseases^26^. Considering countries around the world, very few are on target to achieve the SDG 2030 target. In 2018, unconditional probability of death due to the four main NCDs in 2018 ranged from 4.3% (South Korea) to 31.5% (Sierra Leone), and in the Latin America and Caribbean region from 8.9% (Costa Rica) to 28.4% (Guyana)^27^. Brazil had the largest decrease among major countries in the Region between 2015 and 2018, and rose from the 63^th^ lowest to 58^th^ lowest probability in a global ranking of 186 nations^27^. Many other countries of the Region (e.g., USA and Mexico) had little to no change over this period, and some countries worsened. NCD Countdown 2030 estimated, based on 2010-16 trends in 186 countries and without considering the later disruptions due to COVID-19, that women of only 17 (9.7%) and men in only 15 (8.5%) countries were on target to reach the SGD 3.4.1 goal^21^ ^,^ ^27^. 

Recognition that Brazil will likely not reach its NCD goals nor that put forth in the SDGs is important. Major increases in public health measures to control these conditions seem necessary. The way forward to achieve these goals has been charted internationally but has been incompletely implemented in Brazil and elsewhere. Importantly, it involves the greater use of population-based strategies to control risk factors through actions requiring little personal agency^28^ ^,^ ^29^. Lessening social inequities is another major approach to reaching the goals^30^. Unfortunately, the current political and economic instability, and polarized political climate in Brazil makes implementation of the most effective of these actions, most of which would need to be implanted on a national level, extremely difficult.

In conclusion, premature mortality in Brazil has declined continuously from 1990 to date, with the rate of decline diminishing over time. If the decline continues at its current rate, neither the WHO´s 2025 NCD nor the 2030 SDG goals of reduction in premature mortality will be reached. The unconditional probability of death and the age-standardized mortality rate for the four main NCD groups produce similar estimates of decline, and mirror well decline in mortality from all NCDs. The recent flattening in the rate of decline is worrisome and suggests that greater efforts to control the NCDs are warranted. Investing in measures to regulate risk factors, strengthen prevention and control policies, and reduce inequalities are all essential to placing Brazil back on track in its reduction of NCD disease burden. 
