# Supplementary material for: Premature mortality due to four main non-communicable diseases and suicide in Brazil and its states from 1990 to 2019: A Global Burden of Disease Study
Source: Rev Soc Bras Med Trop. 2022 Jan 28;55(Suppl 1):e0328-2021. doi: 10.1590/0037-8682-0328-2021 (PMC9009436; doi:10.1590/0037-8682-0328-2021)
Supplement: Supplementary file 1 [file 1678-9849-rsbmt-55-s01-e0328-2021-supp1.pdf]

Supplementary Table 1: List of International Classification of Diseases (ICD) codes mapped to the Global Burden of Disease cause list for causes of death.

Cause	ICD 10 codes	ICD 9 codes
Cardiovascular diseases	B33.2, G45-G46.8, I01-I01.9, I02.0, I05-I09.9, I11-I11.9, I20-I25.9, I27.0, I27.2, I28- I28.9, I30-I31.1, I31.8-I37.8, I38-I41.9, I42.1-I42.8, I43-I43.9, I47-I48.9, I51.0-I51.4, I60-I63.9, I65-I66.9, I67.0-I67.3, I67.5-I67.6, I68.0-I68.2, I69.0-I69.3, I70.2-I70.8, I71-I73.9, I77-I83.9, I86-I89.0, I89.9, I98, K75.1	036.4, 391-391.9, 392.0, 393-398.9, 402-402.9, 410-414.9, 416.0, 417-417.9, 420- 423, 423.1-423.9, 424.0-424.3, 424.8, 425.0-425.3, 425.5, 425.7-425.8, 427.0-427.3, 427.6-427.8, 429.0, 430-435.9, 437.0-437.2, 437.5-437.8, 440.2, 440.4, 441-443.9, 447-454.9, 456, 456.3-457, 457.1, 457.8-457.9, 459, 459.1-459.3
Neoplasms	C00-C13.9, C15-C22.8, C23-C25.9, C30-C34.9, C37-C38.8, C40-C41.9, C43-C45.9, C47-C54.9, C56-C57.8, C60-C63.8, C64-C67.9, C68.0-C68.8, C69.0-C69.8, C70- C73.9, C75-C75.8, C81-C86.6, C88-C91.0, C91.2-C91.3, C91.6, C92-C92.6, C93- C93.1, C93.3, C93.8, C94-C96.9, D00.1-D00.2, D01.0-D01.3, D02.0-D02.3, D03- D06.9, D07.0-D07.2, D07.4-D07.5, D09.0, D09.2-D09.3, D09.8, D10.0-D10.7, D11- D12.9, D13.0-D13.7, D14.0-D14.3, D15-D16.9, D22-D24.9, D26.0-D27.9, D28.0- D28.1, D28.7, D29.0-D29.8, D30.0-D30.8, D31-D36, D36.1-D36.7, D37.1-D37.5, D38.0-D38.5, D39.1-D39.2, D39.8, D40.0-D40.8, D41.0-D41.8, D42-D43.9, D44.0- D44.8, D45-D47.9, D48.0-D48.6, D49.2-D49.4, D49.6, K62.0-K62.1, K63.5, N60-N60.9, N84.0-N84.1, N87-N87.9	140-148.9, 150-155.1, 155.3-158.9, 160-164.9, 170-175.9, 180-180.9, 182-183.8, 184.0-184.4, 184.8, 185-186.9, 187.1-187.8, 188-188.9, 189.0-189.8, 190-190.8, 191- 193.9, 194.1-194.8, 200-204.0, 204.2, 205-205.3, 206-206.1, 207-208.9, 209.0-209.1, 209.4-209.5, 210.0-210.9, 211.0-211.8, 212.0-212.8, 213-213.9, 217-217.8, 219.0, 220-220.9, 221.0-221.8, 222.0-222.8, 223.0-223.8, 224-228.9, 229.0, 229.8, 230.1- 230.8, 231.0-231.2, 232-232.9, 233.0-233.2, 233.4-233.5, 233.7, 234.0-234.8, 235.0, 235.4, 235.6-235.8, 236.1-236.2, 236.4-236.5, 236.7, 237-237.3, 237.5-237.9, 238.0- 238.9, 239.2-239.4, 239.6, 569.0, 610-610.9, 622.1-622.2, 622.7
Chronic respiratory diseases	D86-D86.2, D86.9, G47.3, J30-J35.9, J37-J39.9, J41-J46.9, J60-J63.8, J65-J68.9, J70, J70.8-J70.9, J82, J84-J84.9, J91, J91.8-J92.9	135-135.9, 327.2-327.8, 470, 470.9-474.9, 476-476.1, 477-479, 491-493.9, 495- 504.9, 506-506.9, 508-509, 515, 516-517.8, 518.6, 518.9, 519.1-519.4
Diabetes mellitus	E10-E10.11, E10.3-E11.1, E11.3-E12.1, E12.3-E13.11, E13.3-E14.1, E14.3-E14.9	775.1
Suicide	X60-X64.9, X66-X83.9, Y87.0	E950-E959
