# Supplementary material for: Premature mortality due to four main non-communicable diseases and suicide in Brazil and its states from 1990 to 2019: A Global Burden of Disease Study
Source: Rev Soc Bras Med Trop. 2022 Jan 28;55(Suppl 1):e0328-2021. doi: 10.1590/0037-8682-0328-2021 (PMC9009436; doi:10.1590/0037-8682-0328-2021)
Supplement: Supplementary file 2 [file 1678-9849-rsbmt-55-s01-e0328-2021-supp2.pdf]

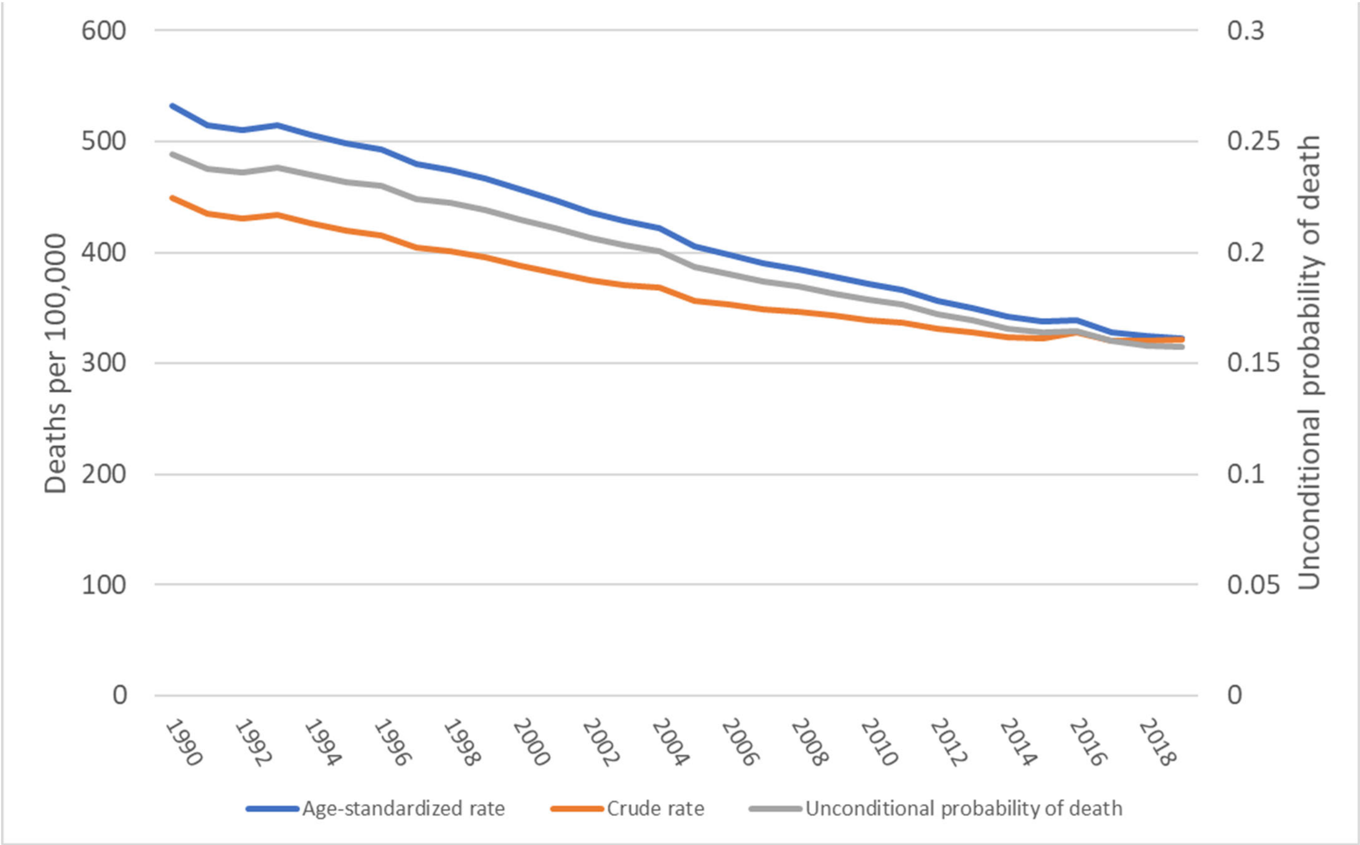


**Supplementary Figure 1:** Comparison of different measures of trends in premature mortality (ages 30 to 69) due to the four main non-communicable diseases in Brazil, 1990-2019. Blue line: Age standardized mortality, Red line: Crude mortality. Grey line: Unconditional probability of death. The standard population is that of 2019.
