# Supplementary material for: Premature mortality due to four main non-communicable diseases and suicide in Brazil and its states from 1990 to 2019: A Global Burden of Disease Study
Source: Rev Soc Bras Med Trop. 2022 Jan 28;55(Suppl 1):e0328-2021. doi: 10.1590/0037-8682-0328-2021 (PMC9009436; doi:10.1590/0037-8682-0328-2021)
Supplement: Supplementary file 4 [file 1678-9849-rsbmt-55-s01-e0328-2021-supp4.pdf]

Supplementary Table 2: Estimated rates and percent declines in age-standardized mortality and the unconditional probability of death from the four main NCDs, ages 30-69, to be achieved based on the assumption that future declines will be equal to those in these metrics over two periods – from 2010 to 2019 and from 2015 to 2019. Brazil.

		Age-standardized mortality		Unconditional probability of death	
		Assuming the decline in rate of		Assuming the decline at rate of	
		2010-2019	2015-2019	2010-2019	2015-2019
	Year	Rate to be achieved*		Probability to be achieved	
	2025	294.2 (277.8; 310.9)	302.5 (277.9; 328.1)	14.5% (13.7; 15.2)	14.8% (13.8; 16.0)
	2030	272.1 (252.3; 293.3)	286.4 (252.2; 324.2)	13.5% (12.6; 14.4)	14.1% (12.6; 15.8)
Target period	Target Goal	Decline to be achieved		Estimated total	Decline to be achieved
2010-2025	25%	-20.8% (-25.2; -16.1)	-18.6% (-25.0; -11.6)	-18.9% (-22.9; -14.6)	-16.9% (-22.8; -10.4)
2015-2030	33%	-19.3% (-25.0; -11.6)	-15.0% (-25.1; -3.4)	-17.6% (-22.8; -12.0)	-13.7% (-23.1; -3.1)

*/100,000
