# Supplementary material for: Premature mortality due to four main non-communicable diseases and suicide in Brazil and its states from 1990 to 2019: A Global Burden of Disease Study
Source: Rev Soc Bras Med Trop. 2022 Jan 28;55(Suppl 1):e0328-2021. doi: 10.1590/0037-8682-0328-2021 (PMC9009436; doi:10.1590/0037-8682-0328-2021)
Supplement: Supplementary file 5 [file 1678-9849-rsbmt-55-s01-e0328-2021-supp5.pdf]

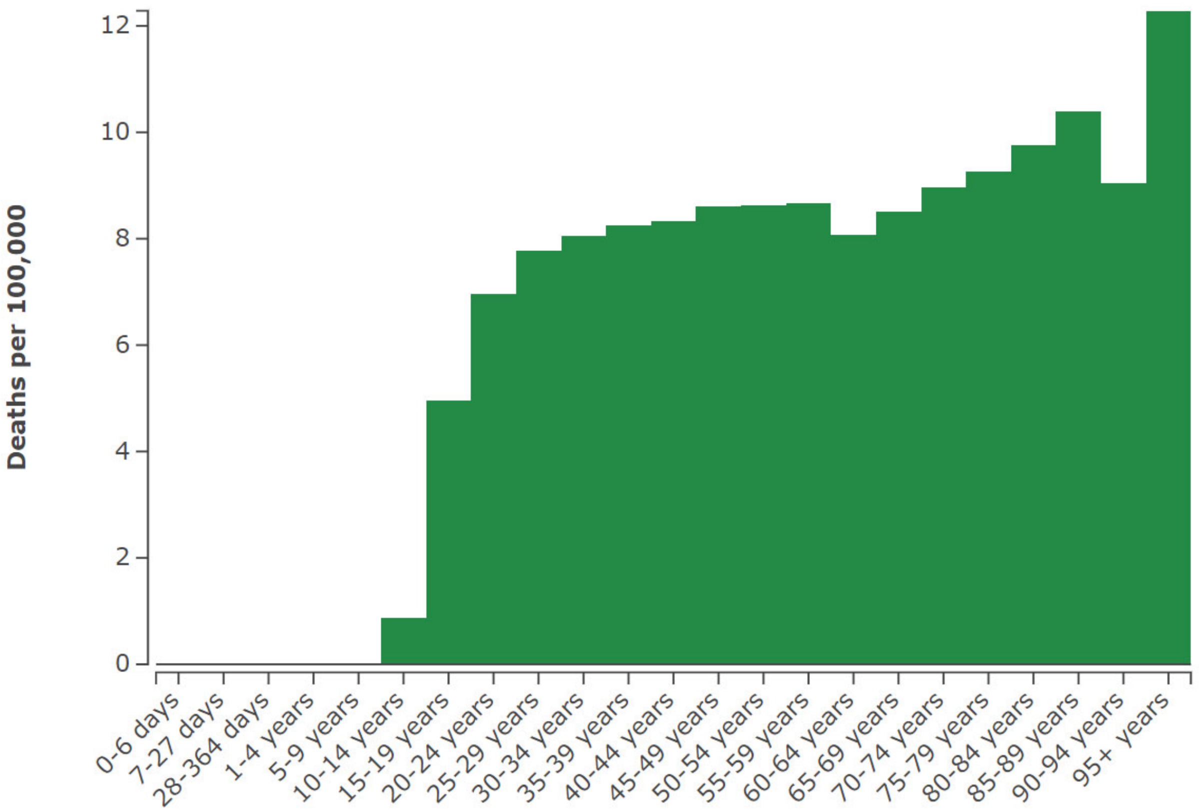

Supplementary Figure 3: Mortality (/100000) across the age span due to self-harm, both sexes, 2019. Brazil. See the specific values of rates of deaths per 100,000 at: http://ihmeuw.org/5gju.
